# Disassembly of self-assembling peptide hydrogels as a versatile method for cell extraction and manipulation†

**DOI:** 10.1039/d4tb01575d

**Published:** 2024-10-17

**Authors:** Cosimo Ligorio, Magda Martinez-Espuga, Domenico Laurenza, Alex Hartley, Chloe B. Rodgers, Anna M. Kotowska, David J. Scurr, Matthew J. Dalby, Paloma Ordóñez-Morán, Alvaro Mata

**Affiliations:** a Biodiscovery Institute, University of Nottingham Nottingham UK a.mata@nottingham.ac.uk; b School of Pharmacy, University of Nottingham Nottingham UK; c Department of Chemical and Environmental Engineering, University of Nottingham Nottingham UK; d Centre for the Cellular Microenvironment, School of Molecular Biosciences, College of Medical, Veterinary and Life Sciences, Mazumdar-Shaw Advanced Research Centre, University of Glasgow Glasgow G11 6EW UK; e Translational Medical Sciences Unit, School of Medicine, Centre for Cancer Sciences, Biodiscovery Institute, University of Nottingham Nottingham UK

## Abstract

Self-assembling peptide hydrogels (SAPHs) are increasingly being used as two-dimensional (2D) cell culture substrates and three-dimensional (3D) matrices due to their tunable properties and biomimicry of native tissues. Despite these advantages, SAPHs often represent an end-point in cell culture, as isolating cells from them leads to low yields and disruption of cells, limiting their use and post-culture analyses. Here, we report on a protocol designed to easily and effectively disassemble peptide amphiphile (PA) SAPHs to retrieve 3D encapsulated cells with high viability and minimal disruption. Due to the pivotal role played by salt ions in SAPH gelation, tetrasodium ethylenediaminetetraacetic acid (Na_4_EDTA) was used as metal chelator to sequester ions participating in PA self-assembly and induce a rapid, efficient, clean, and gentle gel-to-sol transition. We characterise PA disassembly from the nano- to the macro-scale, provide mechanistic and practical insights into the PA disassembly mechanism, and assess the potential use of the process. As proof-of-concept, we isolated different cell types from cell-laden PA hydrogels and demonstrated the possibility to perform downstream biological analyses including cell re-plating, gene analysis, and flow cytometry with high reproducibility and no material interference. Our work offers new opportunities for the use of SAPHs in cell culture and the potential use of cells cultured on SAPHs, in applications such as cell expansion, analysis of *in vitro* models, cell therapies, and regenerative medicine.

## Abstract 2

Peptide hydrogels are gaining much interest as 3D cell-laden matrices for cell culture. Here, a tunable protocol based on a metal chelator is proposed to disassemble peptide hydrogels and extract living cells for downstream biological applications.

## Graphical abstract

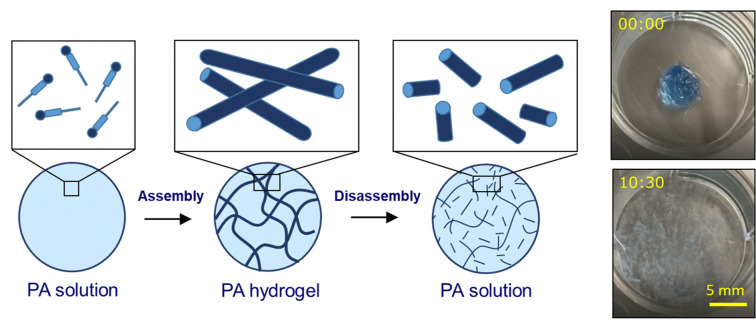

## 1. Introduction

Hydrogels are ideal materials to grow cells, organoids, and microorganisms. Constituted by a water-swollen network of natural or synthetic polymers, hydrogels provide a synthetic microenvironment that can closely resemble structural features of native tissues, facilitating recreation of cell–matrix interactions found *in vivo*. This possibility has inspired different strategies to engineer hydrogels based on natural and synthetic building blocks that can enhance the capacity to control the cellular microenvironment to grow, manipulate, or study cells in culture.

Self-assembling peptide hydrogels (SAPHs) offer the capacity to control the presentation of biochemical cues, recreate ECM-like nanofibrous structures, and modulate cell–matrix interactions.^1^ Moreover, being non-animal derived and chemically-defined, SAPHs have the advantage of high reproducibility compared to organic hydrogels such as Matrigel.^2^ These properties have enabled the use of SAPHs as substrates for 2D and 3D cell culture. For instance, ionic complementary peptides developed by the groups of Zhang^3^ and Saiani^4^ have enabled the creation of rationally-designed scaffolds to grow and differentiate multiple cell types.^2,5–7^ Also, innovative work by Collier and colleagues has showcased SAPHs as immuno-modulatory biomaterials,^8–10^ while Ulijn and co-workers have developed enzyme-responsive spatiotemporal materials for *in vitro* cancer studies.^11,12^ Notably, Stupp and colleagues have pioneered SAPHs based on peptide amphiphiles (PAs) with function-encoding, cell-instructive features to promote cell adhesion,^13,14^ proliferation,^15,16^ and differentiation.^17–20^ This work has inspired others to develop SAPHs to mimic cellular microenvironments^21–23^ or recreate adaptable, antibacterial matrices.^24–26^ Similarly, our group has developed PA-based approaches to engineer SAPHs for cell culture with the capacity to tune structural^27–30^ and signalling^31,32^ properties to grow complex multicellular systems.^33–35^ These examples demonstrate the design versatility and multifunctional capabilities that SAPHs offer for cell culture.

As cell culture substrates become more tunable and versatile, an increasing number of opportunities and applications continue to emerge. For example, cell culture platforms offer the possibility to study fundamental cell behaviours in a controlled manner,^36^ recreate physiological conditions to diagnose disease,^37^ and reconstruct pathological environments *in vitro* to screen new drugs and therapies.^38^ Many cell culture substrates are emerging as highly tunable material platforms for stem cell growth, capable of being shaped and patterned with high precision,^39,40^ injected,^41,42^ or used as depots to deliver pharmaceutical ingredients.^43,44^ Furthermore, cell culture platforms are also critical to expand cell populations for cell therapies,^45^ recombinant production of molecules,^46^ or design of tissue engineering constructs.^47^ These examples demonstrate the diverse uses and widespread impact that new cell culture platforms can have in the fields of biology and biotechnology.

Despite these opportunities, hydrogels are often an end-point in cell culture. This is an important limitation for applications requiring the culture of cells and their extraction in a non-destructive manner with minimal effect on the cells. Current strategies for cell extraction, often involving mechanical or enzymatic methods, present multiple limitations such as incomplete hydrogel degradation, disruption of cellular components, and low cell viability. The possibility of culturing and extracting living cells from 3D hydrogels in a simple and gentle manner would open or enhance opportunities in multiple applications. First, it would facilitate “recycling” and expansion of encapsulated cells, which is advantageous for sensitive, low-proliferative, or expensive cell types. Second, it would enable performance of robust and enhanced biological analyses (*e.g.* qPCR, flow cytometry, single-cell analysis) after encapsulation and retrieval to better assess the effects of drugs or treatments. Finally, the capacity to safely and gently extract expanded cell populations would facilitate the therapeutic use of cells in different kinds of cell therapies in tissue engineering and regenerative medicine.

In this study, we report on a gentle method to disassemble 3D cell-laden SAPHs to safely and effectively retrieve encapsulated cells. Due to the importance of metal ions in the gelation of supramolecular PA hydrogels, we exploited a metal chelator to sequester metal ions responsible for the self-assembly of PAs, inducing a gentle gel-to-sol transition that releases encapsulated cells in a rapid and unaltered manner. We extensively characterised the PA disassembly at multiple size-scales, describing the mechanism and generating insights on the effect of metal chelators on supramolecular peptide disassembly. We demonstrate that our SAPH disassembly method is cell-friendly, allows for full cell extraction within 10 minutes, and enables re-plating of extracted cells for sub-passaging or downstream cell analysis including qPCR and flow cytometry. We envisage that this versatile method will be easily adaptable to other peptide-based systems and broadly used in the fields of peptide science, cell biology, tissue engineering, and regenerative medicine.

## 2. Experimental section

### 2.1. Materials

PAs in lyophilised form were purchased from Cambridge Bioscience, UK. All peptides were purified by HPLC and purity values >95% for every sequence used. Fura-2 pentapotassium salt (code: F1200) was purchased from ThermoFisher UK, ethylenediamine tetraacetic acid tetrasodium salt hydrate or Na_4_EDTA (code: 03701) was purchased from Merck (Darmstadt, Germany). If not stated otherwise, all reagents were purchased from Sigma Aldrich, UK.

### 2.2. PA hydrogel formulations

To create 1% PA-E3 hydrogels, 1 mg of PA-E3 in lyophilised form was dissolved in 70 μL of 10 mM HEPES (pH 7.4) and basified with 10 μL of 0.5 M NaOH until pH 9 to create PA solutions. PA solutions were then mixed with 20 μL of 1 M CaCl_2_ and left undisturbed for 30 minutes to allow complete gelation. PA-E2Y gels were prepared as PA-E3 gels. For 1% PA-K3 gels, 1 mg of PA-K3 powder was dissolved in 80 μL of double deionised water (ddH_2_O) and 20 μL of 1 M NaOH was used to induce gelation. For 1% Fmoc-FF hydrogels, 1 mg of peptide was dissolved in 500 μL DMSO, vortexed for 20 s and added to 500 μL of ddH_2_O. For all the gels tested, gelation was ascertained *via* visual tilting tube tests.

### 2.3. Fura-2 calcium binding test

Fura-2 dye was used to test the ability of Na_4_EDTA to chelate calcium from PA hydrogels. Fura-2 pentapotassium salt was dissolved in ddH_2_O at a final concentration of 1 mg mL^−1^. To create a standard curve of calcium indicator, the Fura-2 aqueous solution was mixed with increasing concentrations of CaCl_2_ in 1 : 1 volumetric ratio and fluorescence emission was measured at 510 nm with excitations at 340 nm (calcium-bound state) and 340 nm (calcium bound state). For testing, 1% PA gels were left to react with 200 mM Na_4_EDTA and diluted 20-fold with ddH_2_O before mixing with the Fura-2 solution. Fluorescence data were collected with a plate reader at room temperature.

### 2.4. Orbitrap secondary ion mass spectrometry (OrbiSIMS)

OrbiSIMS was used to study the effect of Na_4_EDTA on PA molecules. In brief, PA solutions, gels and disassembled gel samples were frozen using the Leica high pressure freezer and introduced into the airlock on the cryostage *via* the Leica vacuum transfer system. The analysis was carried out at −170 °C using a closed-loop liquid nitrogen pumping system (IONTOF GmbH). For the OrbiSIMS analysis, an Ar_3000_^+^ analysis beam with an energy of 20 keV and diameter of 20 μm was used as the primary ion beam. The duty cycle of the beam was set to 4.2% and the gas cluster ion beam (GCIB) current was 245 pA. The analysis was run on an area of 400 × 400 μm using a sawtooth raster mode with sample crater size 497.6 × 497.6 μm. The cycle time was set to 200 μs. Optimal target potential was averaged at approximately ±173.5 V. Depth profiles were collected in both positive and negative polarity, in the mass range of *m*/*z* 75–1125 and the injection time was set to 500 ms. Mass-resolving power was set to 240 000 at *m*/*z* 200. All data analysis was carried out using Surface Lab 7.3 (IONTOF GmbH). Orbitrap data were acquired using a ThermoFisher Orbitrap HF mass spectrometer. Assignments were determined by accurate mass within 2 ppm error of the calculated mass.

### 2.5. ζ-Potential and dynamic light scattering (DLS)

ζ-Potential and DLS measurements were performed to measure PAs’ surface charge and size during assembly and disassembly. PAs were dissolved in MilliQ water at a concentration of 0.1% w/v and basified up to pH 9 using 0.5 M NaOH. Increasing amounts of CaCl_2_ and Na_4_EDTA (0–200 mM as final concentration) were added to PA-E3 solutions to trigger assembly and disassembly. Zeta potential and DLS measurements were acquired at 25 °C in triplicate on three independent samples using a Zetasizer (Nano-ZS ZEN 3600, Malvern Instruments, UK).

### 2.6. Fourier-transform infrared spectroscopy (FTIR)

To check the secondary structure of PA solutions, gels and disassembled gels, FTIR measurements were performed on an Agilent Cary 630 ATR-FTIR analyzer. Samples (20 μL) pipetted on the FTIR machine surface and spectra were recorded as an average of 256 scans with 4 cm^−1^ resolution. Water spectrum (ddH_2_O) was used as a background. For each spectrum a baseline was subtracted and spectra were smoothed using a Savitzsky–Golay filter with a 9-points window in OriginPro software v10.1.

### 2.7. Circular dichroism (CD)

To check the secondary structure of PA solutions and PA after disassembly (PA disassembled) with Na_4_EDTA, circular dichroism (CD) measurements were performed with Chirascan™ circular dichroism spectrometer (Applied Photophysics Limited, UK). Samples (200 μL) were gently pipetted in the quartz cuvette and air bubbles were removed before measurements. Data were collected using quartz cell with 1 mm path length, with 0.5 s per point and 0.5 nm bandwidth in the wavelength range 190–260 nm. Spectra of 10 mM HEPES were used as background for the PA solution sample, while 10 mM HEPES plus 200 mM Na_4_EDTA was used as background for the PA disassembled sample. All CD data were presented as ellipticity and recorded in millidegrees (mdeg). CD spectra were obtained as integration of 3 scans.

### 2.8. Atomic force microscopy (AFM)

AFM was used to image PA-E3 gel disassembly in real time. 1% PA-E3 gels were diluted 20-fold with ultrapure water before drop casting 200 μL of diluted gel on freshly-cleaved mica stubs for 2 minutes. After this time, excess solution was removed with Whatman No. 1 filter paper and samples were exposed to 200 μL of 200 mM Na_4_EDTA aqueous solution. Samples were scanned in fluid at room temperature in TappingMode, using a Bruker Dimension Icon equipped with MLCT probe (Bruker AXS S.A.S., France) with a nominal spring constant (*k*) of 0.07 N m^−1^. Images with scan sizes of either 4 μm × 4 μm were captured at a scan rate of 1 Hz and at a relative humidity of <50%. All AFM images were 1st order flattened prior to analysis using the Gwyddion software v2.62.

### 2.9. Transmission electron microscopy (TEM)

TEM was used to examine the nanostructure of PA-E3 as solution, gel and disassembled gel. All samples were diluted 20-fold with ultrapure water up to 0.05% w/v and mounted on ultrathin carbon grids bar square 400 mesh (CFT400-Cu-UL, Electron Microscopy Sciences, UK). In detail, 20 μL of sample was pipetted on TEM grids for 30 s and excess sample was removed with Whatman No. 1 filter paper. Grids were stained with 2 μL of 2% uranyl acetate for 30 s before washing with 18 μL of ultrapure water. Excess water was then removed with filter paper and samples were air dried for 1 hour at room temperature before imaging. Bright-field transmission electron microscopy was performed on a FEI Tecnai Biotwin-12 TEM running at 100 kV and equipped with a Gatan SIS Megaview IV camera. Fiber length analysis was performed by measuring randomly the lengths of 250 fibres per sample across three independent TEM images using ImageJ software (v1.54j).

### 2.10. Hydrogel disassembly

To quantify the hydrogel weight over time after exposure to Na_4_EDTA, 100 μL of the PA hydrogel was placed in a circular container and exposed to 200 μL of increasing concentrations of Na_4_EDTA (0–200 mM). After every min the Na_4_EDTA solution was gently removed and hydrogel weighed. The remaining mass of hydrogel was calculated as: 100 × *W*_t_/*W*_0_, where *W*_0_ and *W*_t_ were initial and final hydrogel weight, respectively.

### 2.11. Oscillatory shear rheology

Rheology was performed to check the mechanical properties of PA-E3 gels during disassembly. Rheological measurements were performed using an Anton Paar MCR102 rheometer (St Albans, UK) using a 8 mm parallel plate geometry and a 500 μm gap. Samples were prepared as described earlier. Hydrogels were placed onto the rheometer's static bottom plate and the upper plate was moved to the measuring gap size. A solution of 200 mM Na_4_EDTA was slowly pipetted around the gap size to allow infiltration into the PA-E3 gel and an O-ring was placed around the testing area to allow constant exposure to Na_4_EDTA. A time sweep experiment was performed at 37 °C and 1 Hz in the 0.01–100% strain range.

### 2.12. Release of microbeads from disassembled gels

To assess PA gel disassembly at the macroscale, the release of polystyrene beads from PA-E3 gels was assessed. In details, 40 μL of Rhodamine B-coated polystyrene beads (Polybead® Microspheres 3 μm; PolySciences) were mixed with 40 μL of 2% PA-E3 gel before adding 20 μL 1 M CaCl_2_ to get 1 × 10^4^ beads per gel. Beads-containing gels were then exposed to 500 μL of 200 mM Na_4_EDTA solution using a 24 well-plate transwell insert (ThinCert, code: 662638, pore diameter 8 μm). Over time, the solution in the 24 well plate was collected and fluorescence of Rhodamine beads was measured using a fluorescence plate reader (excitation wavelength: 563 nm; emission wavelength: 603 nm). Gels exposed to 1× PBS were used as controls.

### 2.13. Cell encapsulation and cell culture

NIH-3T3 fibroblasts were cultured using DMEM-GlutaMAX^TM^ medium supplemented with 10% v/v foetal bovine serum (FBS) and 1% v/v of antibiotics (100 units per mL penicillin, 100 μg per mL streptomycin, 0.25 μg per mL amphotericin (PSA)). Immortalized human mesenchymal stem cells (HiMSCs, *TERT* and E6/7 immortalized in house^48^) and BM-MSCs (purchased from Promocell©) were cultured in DMEM-GlutaMAX^TM^ supplemented with 10% FBS and 1× PSA. CaCo-2 cells were cultured in DMEM supplemented with 1% PSA and 10% FBS. Hematopoietic stem cells (HSCs) were isolated from human bone marrow and cultured in Iscove's modified Dulbecco medium (IMDM) containing 20% BIT 9500 serum substitute (STEMCELL Technologies), 1% 200 mM l-glutamine (Sigma-Aldrich), 1% 10 mg mL^−1^ penicillin/streptomycin (Invitrogen) supplemented with Flt3L (50 ng mL^−1^), SCF (50 ng mL^−1^) and TPO (25 ng mL^−1^). Cell culture was maintained in a humidified incubator with 5% CO_2_ in air at 37 °C. For cell seeding experiments, cells were passaged at confluency 70–80% with passage *P* < 10 using 1× trypsin-EDTA solution. NIH-3T3 and HiMSCs were seeded at a cell density of 2.5 × 10^6^ cells per mL, CaCo-2 with a density of 0.5 × 10^6^ cells per mL, BM-MSCs as 1 × 10^6^ cells per mL, while HSCs and THP1 were seeded as 0.6 × 10^6^ cells per mL. To get 100 μL of cell-laden gel, 80 μL of basified PA solution was mixed with 20 μL of cell suspension supplied with CaCl_2_ to trigger gelation as described above. Gels were seeded in 96 well-plates and bathed with 100 μL of culture media. Experiments were performed in biological replicates (*n* = 3) per condition.

### 2.14. Cell extraction protocol

To extract cells from PA-E3 gels, culture media was removed from 96 well-plates containing cell-laden gels and replaced with 200 μL of 200 mM Na_4_EDTA solution. Gels were then incubated at 37 °C, 5% CO_2_ for 10 minutes. After incubation, the mixture (PA gels plus Na_4_EDTA solution) was gently pipetted up and down twice before being transferred to a 15 mL tube. Wells were then washed twice with 200 μL of 1× PBS and this was transferred to the 15 mL tube. At this stage, 1× PBS was added to the 15 mL tube up to 10 mL. The falcon tube was inverted 10 times to ensure complete homogenisation and centrifuged at 350 g for 5 minutes. After centrifugation a clear cell pellet was visible at the bottom and collected for further applications (see workflow in Fig. S1, ESI†). For comparison with Na_4_EDTA solutions, cell-laden gels were also disrupted using mechanical and enzymatic routes. For the mechanical methods, 100 μL of PA-E3 gel was exposed to 200 μL of 1× PBS and incubated for 10 minutes at 37 °C, 5% CO_2_ for 10 minutes. After that, gels were gently pipetted up and down until complete solubilization and mixed with 9.7 mL of PBS as above. For the enzymatic route, 200 μL of 1× PBS was replaced with 10 mg mL^−1^ of pronase E (Sigma Aldrich). To count the number of retrieved cells, cell pellets were resuspended with medium, mixed (1 : 1 v/v ratio) with a 0.4% trypan blue (Bio-Rad), and cells were counted using an automated cell counter.

### 2.15. Re-plating and cell viability

To test the ability of cells to grow after extraction from PA-E3 gels, extracted cells were plated on tissue culture plastic at a density of 15 × 10^3^ cells per cm^2^. Cell viability was assessed 48 hours post-plating *via* LIVE/DEAD cytotoxicity assay. In detail, a LIVE/DEAD solution (ThermoFisher Scientific, UK) made by 2 mM of ethidium homodimer-1 and 4 mM of calcein-AM was exposed to plated cells for 30 minutes at 37 °C, 5% CO_2_. After incubation, imaging was performed using a Nikon Plate Widefield fluorescence microscope (emission wavelengths: green for live cells at 515 nm; red channel for dead cells at 635 nm; excitation wavelength: 495 nm). Cell viability (%) was obtained as: 100 × [number of alive cells/total cell number (number alive cells + number dead cells)].

### 2.16. RNA extraction

To measure RNA amount and purity after cell extraction, extracted cells were exposed to 1 mL of TRIzol® for 10 minutes at room temperature and RNA was extracted according to the manufacturer's instructions (ThermoFisher, UK). Extracted RNA was quantified using a Nanodrop 2000 UV spectrophotometer and RNA purity was calculated by measuring the absorbance values at 260 and 280 nm, as previously reported.^49^

### 2.17. Assessment of metabolic activity

Cell metabolic activity before and after PA hydrogel disassembly with Na_4_EDTA was assessed with PrestoBlue® assay (code: A13261, ThermoFisher Scientific). For this step, cell pellets were compared with cells encapsulated in PA-E3 gels and extracted as described in Sections 2.12 and 2.13. Cell pellets and extracted cells were seeded in a 96 well-plate and incubated with a mixture of 90 μL of cell culture media plus 10 μL of PrestoBlue® reagent for 10 minutes at 37 °C and 5% CO_2_. After incubation, fluorescence was measured using a Tecan Infinite® M Plex microplate fluorescence reader (excitation wavelength: 560 nm; emission wavelength: 590 nm).

### 2.18. Quantitative polymerase chain reaction (qPCR)

As proof-of-concept, RNA extracted from CaCo-2 cells was reverse transcribed to cDNA and gene expression was measured as described by Martinez-Espuga.^50^ Data were analyzed according to the 2^−ΔCt^ method, with gene expression normalized to the pre-validated reference gene GAPDH. A list of specific markers was analyzed, namely ANPEP, MUC2, CDH1, and MKi67 (see primer details in Table S1, ESI†).

### 2.19. Flow cytometry on extracted cells

To check whether cell markers were still intact after cell extraction, flow cytometry was performed on extracted hMSCs, HSCs and THP1 cells. After cells were extracted from hydrogels, the cell pellet was resuspended in 100 μL of flow cytometry buffer (PBS supplemented with 0.5% w/v BSA and 2 mM EDTA). To detect proportion of live cells, live/dead staining of MSCs was performed using Zombie NIRTM fixable viability kit (diluted 1 : 200). Live/dead staining of THP1 cells was performed using Sytox^TM^ blue dead cell stain (Invitrogen). HSCs were stained with anti-human antibodies against CD38-PE-Cy7 (1 : 200; ThermoFisher), CD34-PE (1 : 100; ThermoFisher) and Hematopoietic Lineage Antibody Cocktail-FITC (5 μL per sample; ThermoFisher). Unstained cells, full stained cells and fluorescence minus one (FMO) controls were included. Cells were stained on ice for 45 min. Cells were then washed and resuspended in 500 μL flow cytometry buffer and data collected using an Attune® NxT Acoustic Focusing Cytometer (ThermoFisher). Data was analysed using FlowJo software (BD).

### 2.20. Statistical analysis

Data were presented as mean ± standard deviation. All experiments were performed using at least three replicates. Data were compared using unpaired *t* test with GraphPad Prism v10. For statistical comparisons (*) *p*-value < 0.05, while “ns” is used for non-significant differences.

## 3. Results and discussion

### 3.1. Rationale of the study

**Fig. 1:**
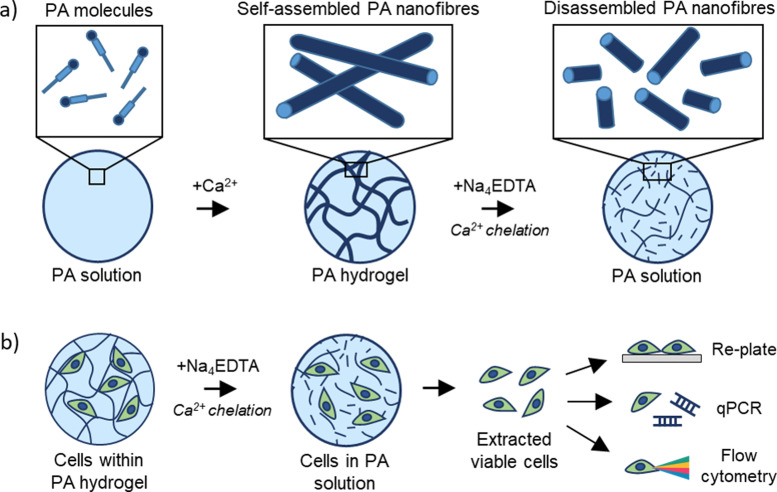
Rationale of the study. (a) Schematics of PA-E3 molecules self-assembling through CaCl_2_ and disassembling through Na_4_EDTA. (b) PA-E3 hydrogels are disassembled to retrieve encapsulated cells for downstream biological analyses.

Polyvalent metal ions such as Mg^2+^,^51–53^ Ca^2+^,^54–56^ and Zn^2+^,^52,57^ play a pivotal role in triggering the self-assembly and tuning the mechanical properties of negatively charged PA hydrogels. Therefore, we hypothesized that the metal chelator tetrasodium ethylenediamine tetraacetic acid (Na_4_EDTA) can be used to induce peptide disassembly through a gentle gel-to-sol transition (Fig. 1). We used PA-E3 (C_15_H_31_CONH-VVVAAAEEE-CONH_2_)-based hydrogels as a model SAPH due to the role of Ca^2+^ ions triggering its gelation and its extensive use as a cell culture hydrogel substrate.^28,33,58,59^ In an attempt to dissect the various stages of PA gel disassembly, we studied the effect of Na_4_EDTA on PA-E3 hydrogels from the nano- to the macro-scale, effectively characterizing molecular assembly and disassembly at different size-scales within the PA solution prior to assembly, PA gel after assembly, and PA gel after disassembly.

### 3.2. Characterisation of PA hydrogel disassembly

#### 3.2.1. Molecular characterisation of PA disassembly

**Fig. 2:**
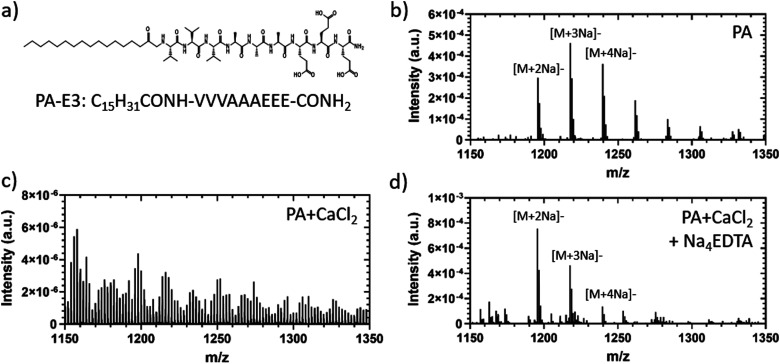
Supramolecular disassembly. (a) Chemical formula of PA-E3 molecules. (b) OrbiSIMS negative polarity spectrum of PA-E3 in solution, (c) as PA gel and (d) as PA gel after disassembly.

To test if the metal chelation could induce a supramolecular disassembly of PA-E3 nanofibres, OrbiSIMS was used to compare PA-E3 solutions, PA-E3 gels (PA + CaCl_2_), and PA-E3 gels plus Na_4_EDTA (PA + CaCl_2_ + Na_4_EDTA). OrbiSIMS combines high spatial resolution secondary ion mass spectrometry (SIMS) with the high mass-resolving power of an Orbitrap analyzer, enabling label-free imaging of molecules.^60–62^ The chemical formula of PA-E3 is shown in Fig. 2a. As observed in the negative polarity OrbiSIMS spectrum for the PA solution (Fig. 2b), sodium adducts of the PA molecular ion were observed at *m*/*z* = 1195.6567 ([M + 2Na]^−^, or C_55_H_93_N_10_O_16_Na_2_^−^), *m*/*z* = 1217.6395 ([M + 3Na]^−^ or C_55_H_92_N_10_O_16_Na_3_^−^), and *m*/*z* = 1239.6214 ([M + 4Na]^−^ or C_55_H_91_N_10_O_16_Na_4_^−^). These sodium adducts were expected to be present, as the PA-E3 solution was basified with sodium hydroxide during formulation (see Experimental section). Upon addition of CaCl_2_, the molecular ion adducts were not detected in the spectrum, which was due to the structures being bound together rather than freely available in the solution (Fig. 2c). The molecular ion adducts were detected again upon addition of Na_4_EDTA to PA gels, indicating disassembly of the bound structure (Fig. 2d). The spectrum of disassembled PAs was not identical to PA solutions, but spectra of disassembled PAs (PA + CaCl_2_ + Na_4_EDTA) and PA solutions (only PAs) shared common ions, such as the molecular ion sodium adducts at *m*/*z* = 1195.6567 ([M + 2Na]^−^, or C_55_H_93_N_10_O_16_Na_2_^−^), *m*/*z* = 1217.6395 ([M + 3Na]^−^ or C_55_H_92_N_10_O_16_Na_3_^−^), and *m*/*z* = 1239.6214 ([M + 4Na]^−^ or C_55_H_91_N_10_O_16_Na_4_^−^). This observation suggested disassembly of PA gels into chemical structures resembling those found in PA solution. At this stage, we also confirmed the ability of Na_4_EDTA to sequester calcium in the presence of PA-E3 nanofibres, as 83.74 ± 12.71% of the calcium ions present in PA + CaCl_2_ was sequestered by the metal chelator as demonstrated by the calcium-binding Fura-2 dye (Fig. S2, ESI†). These results confirm that PA gels can be disassembled by Na_4_EDTA and that PA gel disassembly did not lead to the exact chemical fingerprint of the original PA monomers.

#### 3.2.2. Supramolecular characterisation of PA disassembly

**Fig. 3:**
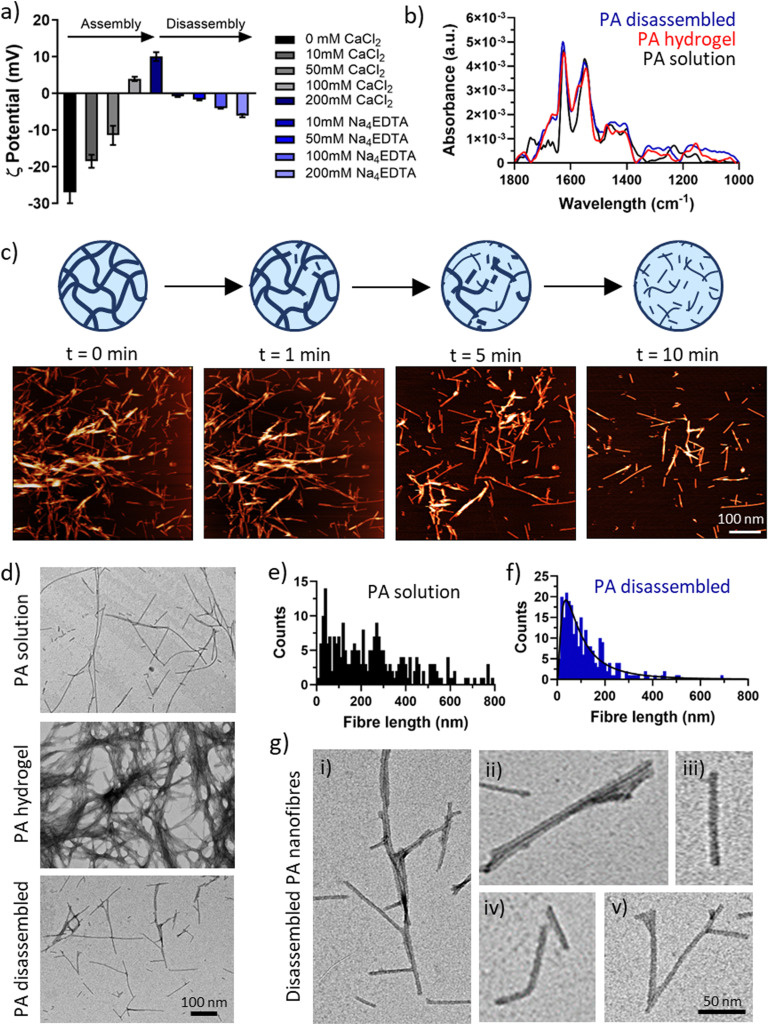
Nano- and micro-scale disassembly. (a) Zeta potential measurements of PA-E3 nanofibres during assembly *via* CaCl_2_ and disassembly *via* Na_4_EDTA. (b) FTIR showing amide regions of PA-E3 in solution before assembly, as gel after assembly and as solution after disassembly. (c) In-fluid AFM shows progressive PA-E3 hydrogel network disassembly when exposed to 200 mM Na_4_EDTA. (d) TEM images of PA nanofibres in solution, in gel and after gel disassembly. Fibre length distribution of PA nanofibres in solution (e) and after gel disassembly (f). (g) Details of (i) disassembled PA nanofibres show (ii) broken nanofibre bundles, (iii) broken individual nanofibre, (iv) bent nanofibre, and (v) intact Y-shaped nanofibre junctions.

To assess PA disassembly by Na_4_EDTA at the supramolecular level, we used zeta potential and DLS measurements. Once dissolved and solubilized with NaOH, PA-E3 forms a clear solution at pH 9. At this pH, the peptide is negatively charged (*ζ* = −27 ± 3.83) given the deprotonation the carboxylic groups side chains of the glutamic acids (p*K*_a_ = 4.25). Increasing amount of CaCl_2_ leads to charge screening and creation of coordination bonds of glutamic acids and calcium. This process resulted in the surface charge of the nanofibres becoming less negatively charged and eventually acquiring a positive charge and increasing their scattering size after addition of 200 mM CaCl_2_ (*ζ* = 10.02 ± 1.21, Fig. 3a). By adding equal concentration of Na_4_EDTA, the surface charge of the PA nanofibers was reduced down to *ζ* = −6.09 ± 0.46 and the scattering size was also reduced, comparable to PA monomers in solution (Fig. S3, ESI†). This finding was in line with previous observations from OrbiSIMS, confirming that chelating calcium causes supramolecular peptide assemblies to disassemble into fragments resembling PA solutions (Fig. 2d). Specifically, this result indicates that, due to calcium chelation, PA disassembly results in PA fragments with reduced surface charge.

To test the effect of chelation on the secondary structure of the PA-E3 nanofibres, FTIR was performed on the PA solution, PA gel (PA + CaCl_2_), and disassembled PA gel (PA + CaCl_2_ + Na_4_EDTA). This step was important to ascertain if the process of disassembly preserved the β-sheet signature of PA nanofibres or whether it induced conformational changes. As shown in Fig. 3b, all the forms of PA supramolecular assemblies presented a typical amide II peak at 1550 cm^−1^, classic of N–H bending and C–N stretching vibrations, and an amide I peak at 1630 cm^−1^ characteristic of β-sheet formation upon assembly.^63–65^ PA solutions, composed by PA monomers arranged into elongated micelles also exhibited a characteristic peak at 1743 cm^−1^, likely originating from the alkyl tail. By comparing the three material forms of PA-E3 (*i.e.* solution, hydrogel, disassembled), it was evident that the spectra of gel and disassembled PA were nearly identical, suggesting that calcium chelation disrupted the PA gels into smaller fragments that still preserved the β-sheet signature of PA nanofibres. A characteristic β-sheet fingerprint was also detected by circular dichroism, with spectra of both PA solutions and PA disassembled showing a positive peak at 203 nm and a negative peak at 220 nm (Fig. S4, ESI†). Coupling together, these results demonstrate that upon addition of Na_4_EDTA, PA gels disassembled into supramolecular structures that preserved negative charge and β-sheet content.

#### 3.2.3. Nano- and micro-scale characterisation of PA disassembly

To further characterize the Na_4_EDTA-induced disassembly process, we investigated the effects of calcium chelation at the PA gel network level. For this experiment, diluted PA gels, were seeded on mica substrates, while a Na_4_EDTA solution was supplied during in-fluid and real-time AFM scanning. As shown in Fig. 3c, under the effect of Na_4_EDTA solution, a dense mesh of at least two layers of PA nanofibres slowly disassembled into smaller domains, leaving a single layer of PA nanofibres on the mica substrates. As shown previously by zeta potential (Fig. 3a), PA gels hold a positive surface charge, due to excess of CaCl_2_ taking part in fibre growth and bundling. On the contrary, mica possesses a negative charge due to exposure of cleaved silicate sheets. Therefore, at *t* = 0 min, PA gels are electrostatically attracted onto mica substrates as shown in Fig. 3c. At *t* > 0 min, we observed that calcium chelation effectively disassembled and liberated PA molecules in solution, which being negatively charged were not further able to deposit on mica. This dynamic event is evident when comparing AFM micrographs over time (Fig. 3c), with PA nanofibres starting to detach from the gel mesh and leaving the scanning area. After 10 minutes of observation, substrate PA coverage dropped by ∼77%, with the thickness of deposited material passing from 47.19 ± 6.09 nm to 13.37 ± 1.22 nm, comparable to the width of the nanofibres. These results reveal that calcium chelation dismantled PA networks of nanofibers (*i.e.*, gels) into individual nanofibres.

To investigate in more detail the nature of the disassembled PA nanofibers, we performed TEM measurements on PA solutions, PA gels, and disassembled PA gels (Fig. 3d). As expected, PA molecules self-assembled in solution into elongated cylindrical micelles with a wide range of fibre length (Fig. 3e). Addition of CaCl_2_ triggered the gelation of PA-E3 molecules into denser networks, with PA nanofibres even bundling into thicker and interpenetrating fibres (Fig. 3d). Addition of Na_4_EDTA disassembled PA gels into individual structures reminiscent of PA nanofibres in solutions, but with a shorter and narrow distribution (Fig. 3f). After disassembly, PA nanofibres were observed as broken structures exhibiting a variety of lengths and morphologies (Fig. 3g,i–iii). Broken individual nanofibres exhibiting bent axes (Fig. 3g,iv) as well as intact peptide Y-shaped junctions were visible (Fig. 3g,v). Y-shaped junctions have been observed in other cylinder-forming peptide systems and arise during peptide self-assembly as peptide lateral association, topological defects or kinetically-trapped branching points.^66–68^ Recently, Cui and co-workers discovered Y-shaped supramolecular assemblies whose junctions may be attributed to fragmented β-sheets with a disrupted internal packing order.^67^ The presence of numerous Y-shaped junctions after disassembly, alongside broken nanofibres, suggests that chelation by Na_4_EDTA is likely to happen preferentially along the surface of individual PA nanofibres, where packing of PA molecules is maximum, rather than occurring in junctions. This interesting observation confirms that Na_4_EDTA disassemble PA gels into PA fragments, more precisely into PA nanofibres preserving Y junctions and cylindrical elongated shapes.

#### 3.2.4. Macroscale characterisation of PA disassembly

**Fig. 4:**
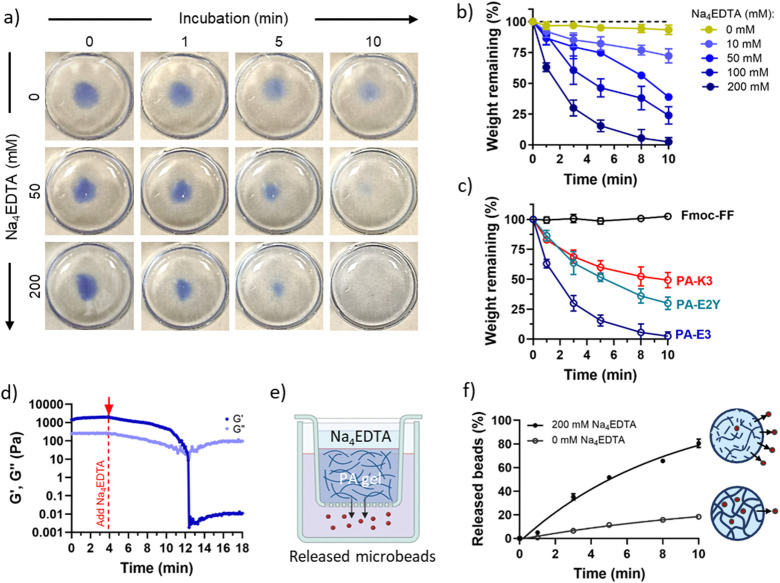
Macroscale disassembly. (a) PA-E3 hydrogels dyed in blue to enhance contrast show progressive disassembly from gel to sol states over time with increasing concentrations of Na_4_EDTA. (b) Weight of remaining hydrogel after exposure to Na_4_EDTA over time. (c) Comparison between PA-E3 and other PA sequences with different epitopes. (d) Effect of Na_4_EDTA on the mechanical properties of PA-E3 gels. (e) Schematics of the release experiment. Rhodamine B-tagged microparticles are embedded in PA-E3 and released upon gel disassembly. (f) Release profile of microparticles with and without Na_4_EDTA.

To assess the effect of disassembly at the macroscale, we investigated changes in the structural properties of the PA gels including shape and mechanical properties. PA-E3 gels assembled with a methylene blue dye started to dissolve into solutions when exposed to increasing concentration of Na_4_EDTA and exposure times (Fig. 4a). This aspect makes the protocol tunable, as systematic modifications of concentration of chelator can induce different degrees of disassembly (as shown in Fig. 4b). For instance, the intact mass of 1% PA-E3 gels quickly decreased over time, completely disappearing after 10 minutes of exposure to 200 mM of Na_4_EDTA (Fig. 4b). A time-lapse video showing PA-E3 hydrogel disassembling over time under the action of 200 mM Na_4_EDTA is shown in the ESI.† To study the effect of PA epitopes on PA gel disassembly, we designed two additional PA sequences to test with the Na_4_EDTA solutions. We know that in the case of PA-E3, PA nanofibres display three glutamic acids (epitope: EEE) to create strong coordination bonds with calcium ions.^52^ In addition to PA-E3, we designed PA-E2Y (epitope: EEY), a negatively charged sequence (*ζ* = −37.7 ± 0.94, Fig. S5, ESI†) where the glutamic acid at the C-terminus was replaced with tyrosine to create weaker π-calcium bonds. As shown in Fig. 4c, we observed that the presence of glutamic acid at the C-terminus led to faster disassembly, compared to tyrosine-displaying nanofibres (∼98% *vs.* ∼70%). This suggests that, in PA-E3, calcium ions were more available for chelation, possibly present in glutamic acid–calcium coordination complexes. To test the effect of ions used for gelation, we also designed PA-K3, a positively-charged PA sequence (*ζ* = 32.93 ± 1.05, Fig. S5, ESI†) displaying lysine residues (epitope: KKK), which undergo gelation with Na^+^ and OH^−^ ions *via* NaOH. As control, with no ions involved, we used Fmoc-FF, which formed hydrogels *via* solvent switch.^69^ Due to the lower affinity of Na_4_EDTA with Na^+^ compared to Ca^2+^ (log *K*_f_ = 10.65 *vs.* log *K*_f_ = 1.86),^70^ PA-K3 exhibited a remarkably reduced capacity to disassemble (∼49%) while, as expected, no disassembly was observed for Fmoc-FF gels (Fig. 4c). This result suggests that PA disassembly can be modulated by changing the propensity of PA nanofibres to bind calcium and by the ionic species used to trigger PA gelation. This features makes the protocol versatile, as it can be applied to different self-assembling peptide hydrogels displaying distinct epitopes (as shown in Fig. 4c).

PA gel disassembly was also evident in terms of gel mechanics. The stiffness of PA-E3 decreased linearly over time after addition of Na_4_EDTA, with a decay of storage modulus of ∼220 Pa min^−1^ (Fig. 4d). Furthermore, the turbidity of PA gels, due to the scattering of light with micrometric gel features in the visible range,^71^ was completely reduced upon exposure to Na_4_EDTA (Fig. S6, ESI†), confirming breaking down of the hydrogel structure into nanometric domains. Similarly, when we encapsulated Rhodamine-tagged polystyrene microbeads (∅ = 3 μm) within PA-E3 gels (Fig. 4e) and exposed the gels to Na_4_EDTA, 80.51 ± 3.57% of the encapsulated beads were released in solution, compared to 18.28 ± 0.95% of beads that passively diffused out from the untreated gels (Fig. 4f). This observation suggested an increase of mesh size for PA-E3 after chelation, with subsequent release of encapsulated microparticles. These results further confirm that Na_4_EDTA is an effective reagent to disassemble PA gels to harvest encapsulated objects.

### 3.3. Biological applications of PA disassembly

#### 3.3.1. Viability of cells extracted from 3D hydrogels

**Fig. 5:**
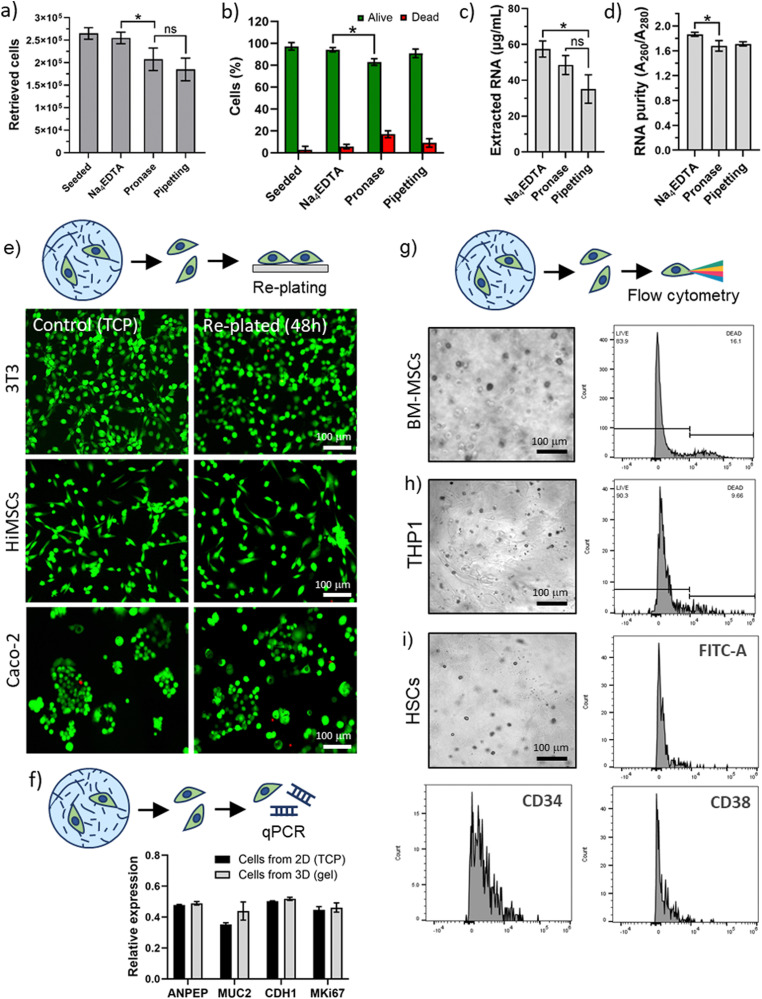
Biological application of PA gel disassembly. (a) Percentage of retrieved cells from 3D PA-E3 gels comparing disassembly *via* Na_4_EDTA with enzymatic (pronase) and mechanical disassembly (pipetting). (b) Viability of extracted cells. (c) Amount and (d) purity of RNA obtained from extracted cells. (e) Re-plating of extracted cells on tissue culture plastic (TCP) and LIVE/DEAD staining of cells extracted from 3D hydrogels, compared to cells grown directly on TCP. (f) RT-qPCR on extracted CaCo-2 cells. (g) Flow cytometry live/dead analysis performed on extracted BM-MSCs, (h) THP1, and (i) HSCs cells. Inset (i) also shows that HSCs retained a CD34^+ve^ phenotype and that a population of CD34^−ve^ stem and progenitor cells remained after cell extraction.

Hydrogel disassembly has a direct application on recovery and manipulation of encapsulated cells. Current approaches of cell retrieval from 3D hydrogels often rely on hydrogel disruption *via* mechanical forces or by using enzymatic degradation, which can be detrimental for cell survival after isolation, as well as leading to incomplete hydrogel disruption. To test the potential of PA disassembly for cell retrieval, we encapsulated 3T3 fibroblasts in PA-E3 and compared PA disassembly *via* Na_4_EDTA with gentle pipetting to enzymatic degradation *via* exposure to pronase E. As shown in Fig. 5a, PA disassembly allowed nearly 96% of cell retrieval from the 3D hydrogels, while enzymatic degradation and pipetting allowed to isolate 78% and 69% of cells, respectively. After PA disassembly with Na_4_EDTA, the entire gel volume was converted into a PA solution, leaving no traces of PA gel and cells in the culture plate. More important, among the cells retrieved with PA disassembly led by Na_4_EDTA, 94.18 ± 2.01% of cells were alive, compared to 82.92 ± 3.02% and 90.86 ± 3.88% for enzymatic and mechanical gel disruption, respectively (Fig. 5b). Similar levels of cell viability were also reported with human immortalised mesenchymal stem cells (94.71 ± 1.38% of alive cells), and human colorectal adenocarcinoma cells (95.97 ± 1.86% of alive cells) extracted from PA-E3 hydrogel with Na_4_EDTA (Fig. S7, ESI†). Moreover, after hydrogel disassembly, cells remained metabolically active, with levels of metabolic activity comparable to those before encapsulation (Fig. S8, ESI†). This result demonstrates that PA disassembly *via* Na_4_EDTA represents a clean and efficient method for cell retrieval from 3D hydrogels.

#### 3.3.2. RNA extraction from isolated cells

Retrieved cells from hydrogels are often exploited for RNA extraction. Here, we used the extracted cells to further extract their RNA and measured its amount and purity. Indeed, high amount of pure nucleic material is essential for gene expression. Absorbance at 260 nm was used to determine the concentration of extracted RNA. Na_4_EDTA and pronase E led to comparable amounts of RNA extracted, while, as expected, pipetting led to the lowest amount of extracted RNA (Fig. 5c). To check the RNA purity, we checked the absorbance ratio *A*_260_/*A*_280_, which should be *A*_260_/*A*_280_ > 1.8–2.1 for pure RNA and that takes into account possible peptide and protein contaminations.^49^ RNA extracted through PA gel disassembly resulted pure (*A*_260_/*A*_280_ = 1.86 ± 0.03), while pronase E and pipetting treatments led to contaminated RNA (*A*_260_/*A*_280_ = 1.68 ± 0.08 and *A*_260_/*A*_280_ = 1.71 ± 0.03, Fig. 5d). We speculate that the incomplete gel-to-sol transition with pronase E and pipetting may lead to carry over of peptide nanofibres after RNA extraction. This result confirms that gel disassembly through chelation has a potential to retrieve alive cells and pure RNA from 3D hydrogels.

#### 3.3.3. Re-plating of extracted cells and gene analyses

Living cells that were extracted from PA gels were re-plated on tissue culture plastic and expanded to check if their functionality was affected by the process of PA disassembly. For this test, we investigated three immortalised cell lines, *i.e.* murine fibroblasts (3T3), human immortalised mesenchymal stem cells (HiMSCs), and human colorectal adenocarcinoma cells (CaCo-2). Cells were chosen as representative of a wide spectrum of cellular “complexity” and differentiation states. Indeed, 3T3 are murine, undifferentiated, and easy-to-use; HiMSCs are of human origin with potency and stem-like properties; while CaCo-2 cells can mimic human tumour biology, are capable of spontaneous differentiation into enterocytes, and are widely-used as a model for intestinal epithelial cells.^50^ Cells were encapsulated in a 3D PA-E3 hydrogel for 1 hour, the gel was then disassembled with Na_4_EDTA and retrieved cells were re-plated in 2D to check their growth over time. As shown in Fig. 5e, cells re-plated after 48 hours were highly viable and with a morphology nearly identical to control cells that did not undergo the process of cell retrieval. As proof-of-concept, human CaCo-2 cells were further examined through RNA extraction and four characteristic markers,^50^ namely ANPEP (as intestinal enterocytes marker), MUC2 (as intestinal goblet cell marker), CDH1 (as epithelial cell marker), and MKi67 (as proliferation marker) were analysed (see full primer details in Table S1, ESI†). Markers had same expression levels of RNA extracted from cells cultured directly on tissue culture plastic, further confirming that PA disassembly leads to clean cells with pure and accessible RNA material (Fig. 5f).

#### 3.3.4. Flow cytometry

Finally, we tested the possibility of performing flow cytometry on cells extracted from 3D PA hydrogels. In this case, having cells with a “clean” surface after extraction is essential, as cells covered by peptides may hinder cell counting and sorting. To ensure the applicability of our method, it is critical to assess its use on hydrogels comprising different cell types exhibiting different levels of sensitivity. For this test, we analysed bone-marrow derived mesenchymal (BM-MSCs) and primary haematopoietic stem (HSCs) cells, as well as leukemic monocytes (THP1). BM-MSCs were chosen for this study as they are immortalised adherent cells widely used in cell therapy and tissue engineering, while THP1 are immortalized suspension cells offering a control to the adherent nature of MSCs. On the other hand, primary cells, such as HSCs, were used as they tend to be more fragile and sensitive to environmental changes compared to immortalised cell lines.

Cells were encapsulated in 3D PA-E3 gels for 1 hour and extracted for flow cytometry analysis. For all cell types, high levels of viability were recorded, further confirming that the PA disassembly process facilitates extraction with minimal effect on the cells. Moreover, analysis of cell surface markers was not hindered. Flow cytometry was performed to assess viability of the BM-MSCs and THP1 cells (Fig. 5g and h) and revealed a 70–80% viability (78.36 ± 5.40% for BM-MSCs and 89.61 ± 0.98% for THP1), while HSCs were successfully analysed by detection of characteristic lineage surface markers (*e.g.* FITC-A, CD34 and CD38, Fig. 5i and Table S2, ESI†). We observed that the HSCs, isolated originally using CD34, retain a large population of CD34^+ve^ cells. The stem (long-term and short-term) and early progenitor population of HSCs were CD38^−ve^ and we observed that many of the CD34^+ve^ cells did not express CD38. This suggests that the clinically useful, stem and progenitor compartment of the CD34 population remains intact after encapsulation and release. Moreover, this result was obtained with primary HSCs, which represents a particularly delicate population of cells.^72,73^ Overall, these results confirm that our PA disassembly process is a clean and cell-friendly method for the extraction of different kinds of cells, including delicate cells such as primary cells.

## 4. Conclusions

In conclusion, we have shown that a metal chelator can be effectively used to disassemble PA hydrogels in a versatile, rapid, and gentle manner suitable for the culture, retrieval, and analysis of cells. The underlying design feature of our process relies on enabling the interaction of the metal chelator with the ions involved in PA assembly, gently breaking down the PA hydrogel network into fragments of self-assembled peptides and resulting in gradual softening and solubilization. This method is rapid, cell friendly, can be easily adaptable to other SAPHs, and opens a number of opportunities. For instance, PA hydrogels used as cell carriers in cell therapies could be selectively disassembled to release encapsulated cargoes for delivery or cell engraftment. Additionally, as demonstrated in this study, PA hydrogels employed as 3D matrices for mammalian cell growth can be disassembled allowing cell extraction, cell expansion, characterization, and manipulation. We envisage that our protocol will facilitate overcoming current limitations of applications relying on cell culture and will benefit fields such as material science, cell biology, and bioengineering.

## Conflicts of interest

The authors declare no conflict of interest.

## Supplementary Material

TB-012-D4TB01575D-s001

TB-012-D4TB01575D-s002

## Data Availability

The data supporting this article have been included as part of the ESI.†
