# Supplementary material for: Disassembly of self-assembling peptide hydrogels as a versatile method for cell extraction and manipulation
Source: J Mater Chem B. 2024 Oct 17;12(46):11939–52. doi: 10.1039/d4tb01575d (PMC11502993; doi:10.1039/d4tb01575d)
Supplement: TB-012-D4TB01575D-s001 [file TB-012-D4TB01575D-s001.pdf]

Supplementary Information (SI) for Journal of Materials Chemistry B.
This journal is © The Royal Society of Chemistry 2024

Supplementary Information

Disassembly of Self-Assembling Peptide Hydrogels as a Versatile Method for Cell Extraction and Manipulation

Cosimo Ligorio[1,2,3], Magda Martinez-Espuga[1,2], Domenico Laurenza[1,2], Alex Hartley[1,2], Chloe B. Rodgers[4], Anna M. Kotowska[2], David J. Scurr[2], Matthew J. Dalby[4], Paloma Ordóñez-Morán[5], Alvaro Mata[1,2,3]*

Affiliations:
[1]Biodiscovery Institute, University of Nottingham, Nottingham, UK
[2]School of Pharmacy, University of Nottingham, Nottingham, UK
[3]Department of Chemical and Environmental Engineering, University of Nottingham, Nottingham, UK
[4]Centre for the Cellular Microenvironment, School of Molecular Biosciences, College of Medical, Veterinary and Life Sciences, Mazumdar-Shaw Advanced Research Centre, University of Glasgow, Glasgow G11 6EW, UK
[5]Translational Medical Sciences Unit, School of Medicine, Centre for Cancer Sciences, Biodiscovery Institute, University of Nottingham, Nottingham, UK

Corresponding author:
Alvaro Mata
School of Pharmacy and Department of Chemical and Environmental Engineering,
University of Nottingham, Nottingham NG7 2RD, UK
Email: a.mata@nottingham.ac.uk

Supplementary Figures

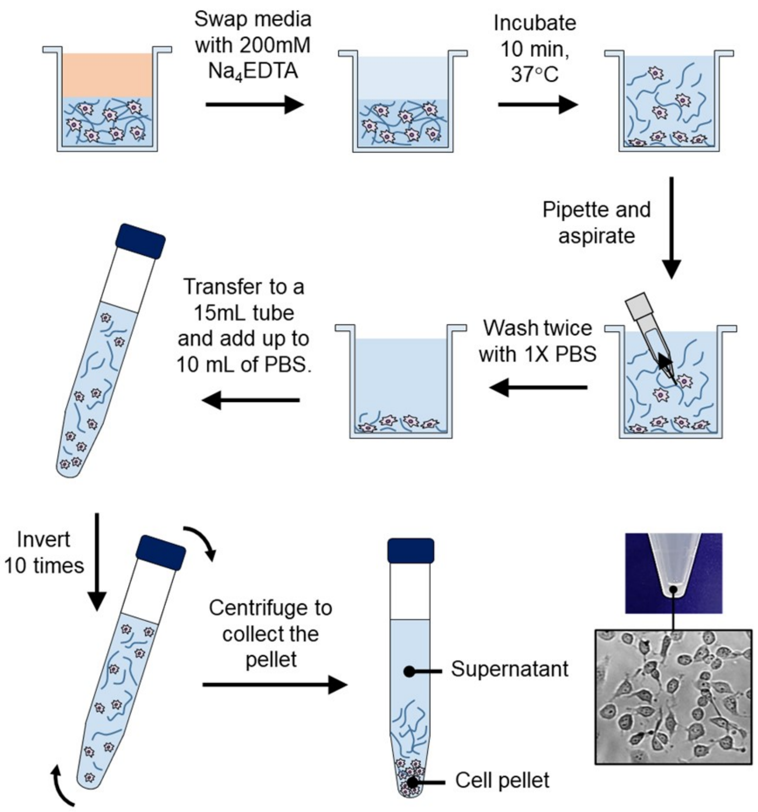

Figure S1. Schematics of the disassembly protocol to retrieve cells from 3D PA hydrogels.

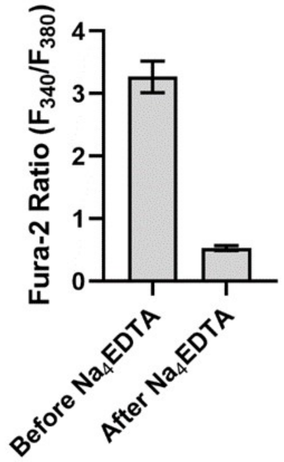

Figure S2. Amount of calcium ions present in PA gels before and after addition of Na_4EDTA.

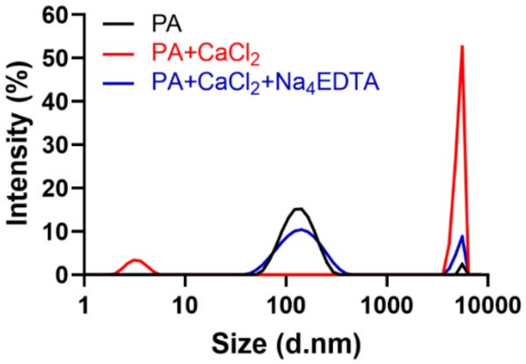

Figure S3. Dynamic light scattering shows size distribution of PA nanofibres in solution (black line), as gel (red line) and as solution after effect of Na_4EDTA (blue line).

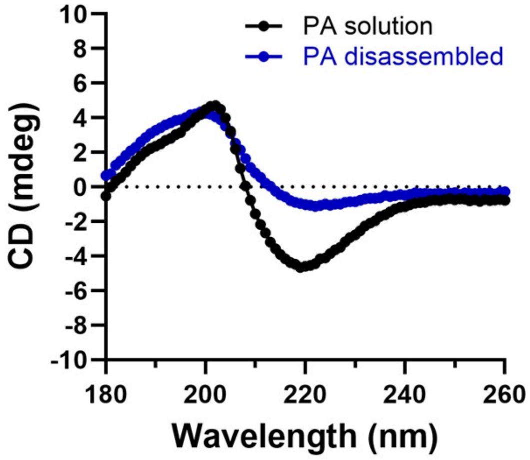

Figure S4. Circular dichroism spectra of PA solution (black line) and PA gel disassembled with Na_4EDTA (blue line).

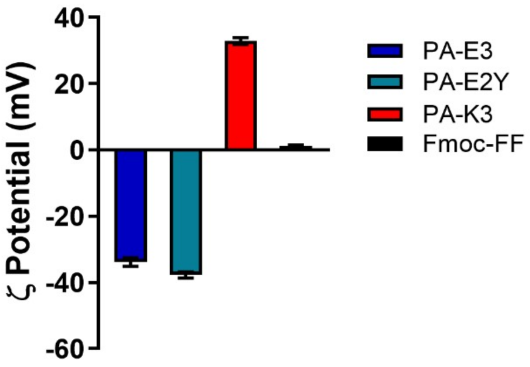

Figure S5. Zeta potential values for the negatively-charged (PA-E3, PA-E3Y), positively-charged (PA-K3) and neutral (Fmoc-FF) peptides tested.

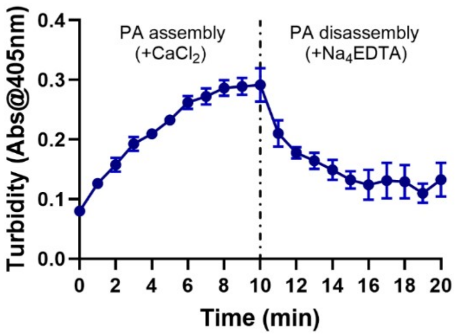

Figure S6. Turbidity measurements of PA solution during assembly and disassembly.

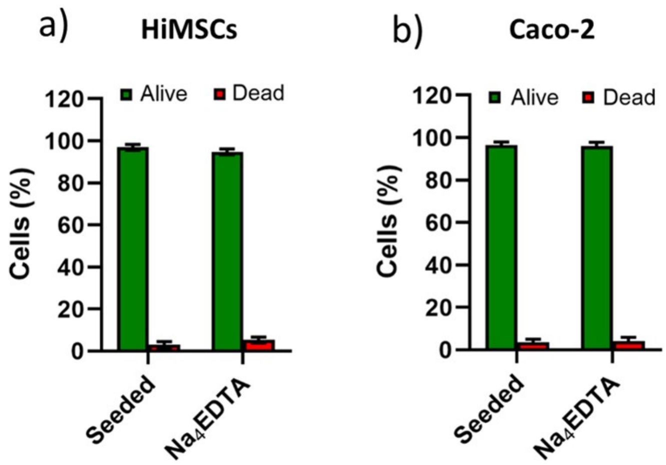

Figure S7. Percentages of alive and dead cells before and after PA-E3 hydrogel disassembly by Na_4EDTA. (a) Cell viability of seeded HiMSCs and extracted HiMSCs. (b) Cell viability of seeded and extracted Caco-2 cells.

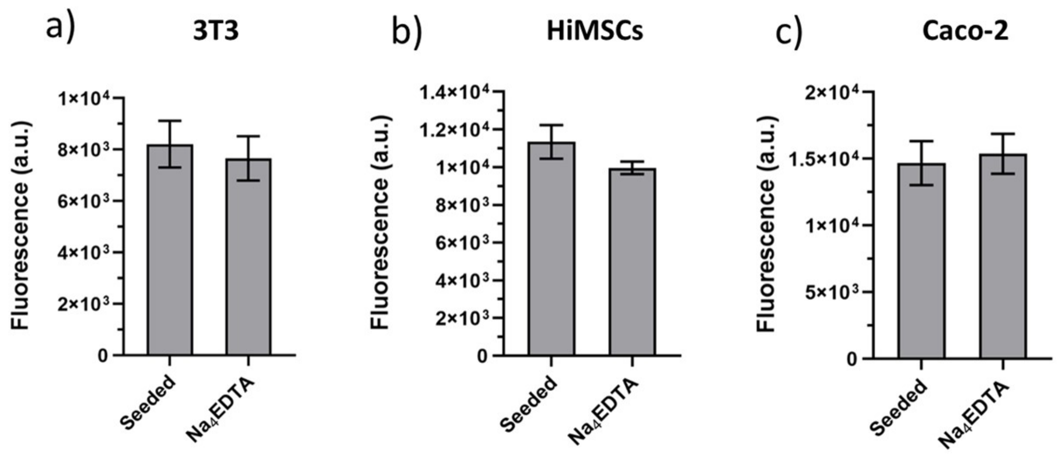

Figure S8. Metabolic activity assessed by PrestoBlue assay for (a) 3T3, (b) HiMSCs and (c) Caco-2 cells before and after PA hydrogel disassembly by Na_4EDTA.

Table S1. Primers used for qPCR analysis of CaCo-2 cell gene expression.

Human gene	Forward primer	Reverse Primer
ANPEP	CATTATGACACACCCTACCCACT	CTCATGAGCAATCACAGTGACC
MUC2	ACCCGCACTATGTCACCTTC	GGACAGGACACCTTGTCGTT
CDH1	GGTGCTGAGGATGAAAAGGGT	GGCAGTGTCTCTCCAAATCCG
MKi67	AGGGAATGAAAGTGCGTGGA	GCTTCTGCTTGGGCTTCTTT
GAPDH	GAGTCAACGGATTTGGTCGT	TTGATTTTGGAGGGATCTCG

Table S2. Detailed proportions of HSC population after flow cytometry. Average percentages of positive and negative cells (HSC %) were obtained from 4 independent experiments (n=4).

	**Lineage**			**CD34**			**CD38**		
	Positive	Negative	SD	Positive	Negative	SD	Positive	Negative	SD
HSC %	19.05	80.95	±8.55	8.04	91.96	±4.16	1.43	98.56	±0.28
